# Neurophysiological Basis of Sleep’s Function on Memory and Cognition

**DOI:** 10.1155/2013/619319

**Published:** 2013-01-01

**Authors:** Rebecca M. C. Spencer

**Affiliations:** Department of Psychology and Neuroscience and Behavior Program, University of Massachusetts, Amherst 419 Tobin Hall, 135 Hicks Way, Amherst, MA 01003, USA

## Abstract

A wealth of recent studies support a function of sleep on memory and cognitive processing. At a physiological level, sleep supports memory in a number of ways including neural replay and enhanced plasticity in the context of reduced ongoing input. This paper presents behavioral evidence for sleep’s role in selective remembering and forgetting of declarative memories, in generalization of these memories, and in motor skill consolidation. Recent physiological data reviewed suggests how these behavioral changes might be supported by sleep. Importantly, in reviewing these findings, an integrated view of how distinct sleep stages uniquely contribute to memory processing emerges. This model will be useful in developing future behavioral and physiological studies to test predictions that emerge.

## 1. Introduction

There is a pervasive belief that sleep is a period of physical and mental inactivity and this inactivity allows us to be “refreshed” upon waking. Perhaps for this reason, we often dismiss the elementary mistakes made by sleep-deprived new parents, the emotionality of a napless child, and the poor performance of a jet-lagged sports team. In 1959, radio personality Peter Tripp provided the ultimate test of sleep deprivation by staying awake for 201 hrs. While his record would be surpassed, this highly public stunt (in a glass booth in New York’s Times Square) left a lasting record of the effects of extreme sleep deprivation. After 72 hrs, Tripp began hallucinating. Emotionally, he was depressed. Eventually he became incoherent [1]. Likewise, individuals with fatal familial insomnia, who gradually lose the ability to sleep, exhibit cognitive dysfunction in conjunction with developing symptoms [2].

Cognitive dysfunction following sleep deprivation suggests a role of sleep in preparing for subsequent performance. Moreover, brain activity during sleep is indicative of cognitive processing taking place in the sleeping brain (e.g., [3]). An emerging area of research, sleep cognitive neuroscience, has provided ample evidence that this brain activity is functional. Intervals with sleep protect [4] and, in some cases, enhance [5] memory in healthy individuals. For example, delayed recall on many memory tasks is more accurate if the intersession interval following learning contained sleep relative to recall following an equivalent interval spent awake [4, 6, 7]. Such benefits are thought to, at least in part, stem from consolidation of recent memories and episodes. Consolidation is a process by which memory storage becomes stronger and more efficient. Sleep-dependent memory consolidation refers to the majority of consolidation occurring specifically over sleep, presumably to minimize disruption from ongoing encoding [8]. Sleep also benefits other cognitive process such as creativity [9], insight [10], and decision making [11]. For example, decision making is impaired following sleep deprivation [12] and enhanced following sleep [11].

Many authors have reviewed the behavioral evidence supporting the role of sleep in memory and cognition in humans (e.g., [13, 14]). Others have reviewed neurophysiological processes occurring during sleep, including hippocampus-based processes that likely reflect memory function (e.g., [15, 16]). However, few recent papers have integrated human behavior with neurophysiological results, which largely emerge from animal research. To be fair, such a review may be premature, given that this integration requires speculation and the assumption that human and animal sleep are similar enough to be inferred across models. Nonetheless, we commence with such a review and a speculative model in order to provoke both sides, the animal physiologists and human behaviorists, to consider how they might relate and, in doing so, to identify collective gaps.

## 2. Neurophysiology of Sleep

From 1916 to the mid-1920s, an epidemic spread throughout Europe and North America. Because of the prominence of sleep symptoms associated with the disease, particularly sleep in unusual positions, it was colloquially referred to as “sleeping sickness” [17]. Upon providing the first medical description, Baron Constatin von Economo named the disease encephalitis lethargica [18]. From histology of the encephalitis lethargica brains, von Economo identified a lesioned area, between the midbrain and the diencephalon, as critical to arousal. In fact, this region is centered to both a wake-promoting pathway and a sleep-promoting pathway [19].

Activation of sleep-promoting pathways eventually yields sleep. However, sleep is not homogenous. Rather, the brain cycles through a number of physiological states over the typical sleep period. Adult humans cycle through these states at a rate of 90 mins per cycle [20]. Cycles are shorter in human infants (approximately 45 mins) [21] and small animals (e.g., 10 mins in rats) but are longer in larger animals (e.g., >100 mins in elephants) [20].

In humans, healthy adult sleep typically begins in non-rapid eye movement (NREM) sleep. The earliest stage of NREM sleep, stage 1 (NREM-1), is evident by a transition from the wake-like alpha waves (8–13 Hz) to the theta waves (4–7 Hz), characteristic of early sleep. This transition is often referred to as the hypnogogic state.

Stage 2 of NREM (NREM-2) sleep follows and is characterized by the appearance of K-complexes and sleep spindles on a background of theta activity. K-complexes are composed of a brief negative sharp wave followed immediately by a positive inflection, taking place within .5 secs [22]. They are generated broadly throughout the cortex and reflect a cortical downstate (i.e., neural inactivity) [23]. K-complexes can be induced by an auditory stimulus and thus are associated with sleep maintenance [24].

Along with K-complexes, NREM-2 is marked by sleep spindles. Sleep spindles are brief bursts (approximately .5 secs) of very high frequency waves (11–16 Hz) [22]. Like K-complexes, spindles have also been associated with sleep maintenance [25, 26] yet physiologically spindles and K-complexes are quite distinct. GABAergic activity in the reticular nucleus of the thalamus underlies the generation of sleep spindles which then spread to the thalamocortical system [27]. Spindles may be uniform but emerging evidence suggests a distinction between “fast” and “slow” sleep spindles. Fast sleep spindles are 13–16 Hz in frequency and are associated with activation in the mesial frontal cortex, hippocampus, and sensorimotor processing areas (pre- and postcentral gyrus and supplementary motor area). Slow spindles have a frequency of 11–13 Hz and are associated with superior frontal gyrus activity [27, 28].

While the majority of spindles are fast spindles and found in NREM-2, slow spindles are also found in stage 3 of NREM sleep, known more commonly as slow wave sleep (SWS). SWS is unique because, as the name implies, background EEG slows to .5–2 Hz [22], or delta waves. SWS spindles occur within slow oscillations. Slow oscillations, which are likely distinct from the visible delta waves [29], represent widespread alternation between depolarized “up-states” and hyperpolarized “down-states” [30].

Early in the night, for humans, SWS is followed by brief bouts of rapid eye movement (REM) sleep. REM bouts lengthen over the night as SWS is replaced by NREM-2. REM’s discovery is another noteworthy tale in the history of sleep research. Eugene Aserinsky, a graduate student under Nathanial Kleitman, devised a way to record eye movements during sleep with the intent to study blink rate at sleep onset. He attached the electrodes to the scalp and face of his 8-year-old son, Armond, an opportunistic research subject. Late in the night, Aserinsky observed wake-like EEG and eye movements on the record. Thinking his son had awoken, the father checked on his son only to find him fast asleep. This observation marked the discovery of REM sleep [31] and what is claimed to be the birth of modern sleep science [32].

REM sleep is characterized by rapid ocular saccades and muscle atonia. EEG, with a frequency of 30–80 Hz, is almost indistinguishable from wake EEG [22]. This high level of brain activity is particularly evident in the thalamus, anterior cingulate cortex, parietal operculum, and amygdala and has been associated with the vivid and imaginative dreams that are typical in this sleep stage [33]. In contrast to this, dreams are unremarkable when awoken from NREM sleep, with dream reports often resembling explicit recent memories [34]. This is consistent with NREM brain activity in parahippocampal gyrus [3], an area of the brain associated with memory encoding.

The neurochemical amalgam also differs greatly between NREM and REM sleep. On the one hand, noradrenergic activity, although lower during sleep than wake, is greater during SWS than REM sleep [35]. On the other hand, acetylcholine levels are low during SWS and high during REM, close to waking levels [36]. Acetylcholine has a known role in memory formation during waking and it has been proposed that high levels of acetylcholine suggest a memory function of REM sleep [37, 38].

While this section provides only a brief review of the neuroanatomical and neurochemical states (for more detail see [35, 39]), the physiological potential for one or more cognitive processing steps to take place during sleep is nonetheless evident. Activity in the medial temporal lobe during SWS and in the amygdala and anterior cingulate cortex during REM sleep hints at this cognitive function. Moreover, these dramatic differences in brain activity across sleep stages along with neurochemical distinctions emphasize that sleep cannot be considered singly. Rather, specific neurophysiological events are likely to underlie specific changes in cognitive functions associated with sleep.

## 3. Sleep-Dependent Memory Consolidation

A pioneer of cognitive psychology, Hermann Ebbinghaus, is regarded for his experimental studies of memory. In 1885, in his seminal publication “Memory: A contribution to experimental psychology” [40], he wrote that the “least satisfactory” of his results was a data point on the forgetting curve (a plot of the number of learned syllables forgotten over time) which did not fall on the line defined by the other data points. In retrospect, it seems that Ebbinghaus made the first observation of the benefit of sleep on memory that errant data point reflected reduced forgetting following an interval that contained sleep.

Interest in Ebbinghaus’s observation emerged very briefly in 1924 when John Jenkins and Karl Dallenbach at Cornell University deliberately compared “obliviscence” (i.e., forgetting) over sleep and wake intervals. In doing so, Jenkins and Dallenbach [41] replicated Ebbinghaus, reporting reduced forgetting over sleep relative to wake. Specifically, participants recalled twice as many of the learned syllables following sleep as they did following wake.

In the past decade, a number of studies have replicated Ebbinghaus’s observation with more participants than Jenkins and Dallenbach (who had only two) and more scrupulous control conditions. Collectively, these studies support a role of sleep in memory consolidation. In its simplest form, sleep-dependent memory consolidation can be seen by studies examining the change in recall following an interval with sleep relative to an interval with wake. For instance, one may learn a list of semantically unrelated word pairs in the morning and recall them 12 hrs later (e.g., 8 am to 8 pm) following a daytime interval spent awake, or one might learn the list in the evening and recall the word pairs 12 hrs later following an interval primarily spent in overnight sleep (e.g., 8 pm to 8 am). Recall is superior in the latter condition, following an interval with sleep (e.g., [4, 7]).

Of course, attention or other cognitive processes necessary to perform well on the task may be at their best in the morning and, as such, superior recall following sleep relative to wake may be a circadian, or time of day, effect. Ours [4, 7] and other [42-44] studies have ruled out this alternative explanation. For instance, immediate recall (which takes place in the morning for the “wake group” and in the evening for the “sleep group”) does not differ for the two groups, suggesting that time-of-day does not alter performance on this task [42]. Moreover, other studies have used a nap paradigm. By comparing recall on a similar task after a mid-day nap with recall following an equivalent mid-day interval spent awake, there is no circadian difference in the time of encoding or recall and yet the sleep benefit remains [44, 45].

Similar benefits have been observed on a wide range of learning tasks. Consider a visuospatial learning task that requires learning the locations of specific items in a matrix, similar to the children’s game of “Memory” (also known as “Concentration”). Like word-pair learning, this task is considered to be largely hippocampus dependent [46], relying on the same spatial learning capabilities that make this structure enlarged in spatial experts like London taxi drivers [47, 48]. Performance on this visuospatial learning task shows similar sleep benefits to those seen in word-pair learning: participants recalled the location of more items following sleep than following an equivalent period spent awake [49, 50].

Sleep’s role in memory consolidation is observed throughout development. Children with sleep disorders, such as sleep apnea [51], narcolepsy [52], and excessive daytime sleepiness [53], have impairments in memory and daytime function. Moreover, memory consolidation is greater over intervals with sleep compared to intervals with wake even at a young age. This has been seen in studies of adolescents [54] and young children [55, 56]. For example, Wilhelm and colleagues [56] found that children, 6–8 yrs of age, were able to recall more word pairs following an interval with sleep than after an equivalent interval of wake. This benefit of sleep was nearly equivalent for the 6–8 yr old children and young adults (average of 26 yrs). We have demonstrated that mid-day naps in preschool children (3–5 yrs) also serve a memory function. When children learn a matrix of locations in a visuospatial task in the morning, memory is superior in the afternoon following a mid-day nap compared to memory following an equivalent interval awake [57]. This result suggests that mid-day sleep is equally important as overnight sleep at least at this young age.

Sleep-dependent memory consolidation is not unique to humans and, in fact, has been observed in a range of nonhuman models from the miniscule fruit fly (*Drosophila*) to our nearest neighbors, the great apes. *Drosophila*, which is an ideal model system due to their known genetic make-up and relatively simple nervous system, consolidate its memories over sleep. To demonstrate this, Donlea and colleagues [58] modified *Drosophila* to express temperature-gated channels downstream from the dorsal fan-shaped body, an area associated with sleepiness in *Drosophila*. These flies received massed training on a courtship protocol, a probe of long-term learning that takes advantage of the fact that male flies who unsuccessfully court female flies will subsequently reduce courtship attempts with receptive virgin female flies [59, 60]. Following training, the temperature-gated channels were activated through a temperature change, thereby inducing sleep [58]. After sleep, long-term memory for the training experience (i.e., reduced courtship) was present whereas no such memory for the courtship training was evident when sleep was not induced.

Sleep also benefits bird song learning and discrimination. Following exposure to a tutor’s song, juvenile zebra finches reproduce the tutor song, an important step in sensorimotor development. Shank and Margoliash [61] found that incorporation of the tutor song was the greatest following intervals containing sleep. Likewise, sleep is beneficial to discrimination learning in adult starlings. Brawn et al. [62] demonstrated this using a go/no-go task where one song segment served as a “go” cue and responses were rewarded with food access while another song segment served as a “nogo” cue and responses to it were punished with an interval of lights out. Discrimination of the two song segments improved significantly over sleep but no change in discrimination ability was found following an equivalent interval spent awake.

The largest nonhuman animals in which sleep-dependent memory consolidation has been studied are the great apes. Chimpanzees, bonobos, and orangutans were tested on their ability to remember the location of a food reward that was placed under one of three cups [63]. Although correct responses steadily decreased over waking intervals of 1, 2, 4, or 8 hrs, recall was largely unchanged over 12 and 24 hr intervals, intervals that contained sleep. This suggests that sleep provides, at minimum, a protective service to memories in the great ape family. Moreover, when animals were given an interference trial before recall of the food reward location, performance remained accurate if sleep occurred following learning. Performance was impaired by the interference trial for those animals that stayed awake following learning. This latter result suggests that sleep’s role was not merely through passive protection of the memory but played an active role, resulting in a more stable memory that was resistant to interference.

In summary, sleep-dependent consolidation is a function of sleep observed across species and from early development into adulthood. With the wealth of evidence in support of this function of sleep, it is useful to turn to understanding how memories are consolidated in the brain and why this takes place during sleep.

## 4. Neurophysiology of Sleep-Dependent Memory Consolidation

Multielectrode recordings in the hippocampus of rodents have provided insight into the mechanism underlying the beneficial effects of sleep on memory. By recording in the hippocampus while an animal explores a maze, a “brain map” of space is revealed. During exploration, the pattern of neural firing in hippocampal CA1 and CA3 cells, known as “place cells,” is predictable as these cells exhibit an increased firing rate when the animal is in a particular location in space [64]. Wilson and McNaughton [65] observed that place cells that fired together during waking exploration tended to also fire together during subsequent sleep. Subsequently, Skaggs and McNaughton [66] found that not only was the same neural ensemble active, but cells were reactivated in the same order during sleep as they were during waking behavior.

Evidence of a role of neural replay on memory formation has also been seen in human research. In one such study [49], participants performed a visuospatial task requiring learning of a matrix of images. learning occurred either in the presence or absence of an odor. The experimental odor was represented during subsequent SWS for a subset of the participants. Rasch and colleagues found superior recall following sleep when the odor present during learning was represented during sleep. Similarly, Rudoy and colleagues [50] had participants learning a visuospatial task in which each image was paired with a specific sound. Half of these sounds were represented during subsequent SWS. Delayed recall was greater for those items for which the associated sound was represented during sleep relative to those items for which the associated sound was not replayed. Importantly, presentation of learning cues during SWS is associated with increased hippocampus activation during sleep [49].

It is hypothesized that the representation of learning cues during sleep primes certain memories to be preferentially replayed. Supporting this assumption, Bendor and Wilson [67] demonstrated that representing learning-related cues during sleep biases hippocampal replay. Rats were trained to run to the right or left of a track depending on which of two sounds were presented at the start of the trial. As expected, place cell ensembles associated with the task were reactivated during sleep. Adding to this, when one of the sounds was presented during sleep, place cells associated with the particular tone played were observed to be more active than those associated with the tone paired with the other direction.

Reactivation of neural “songs” associated with waking experiences (i.e., replay) may be a mechanism underlying sleep-dependent consolidation. At its simplest, by replaying a sequence of firing, the memory may become stronger just as one might learn all the words to a favorite movie by replaying the flick over and over. However, most studies of neural replay are based on well-learned behaviors, lacking direct support for replay’s role in new memory consolidation.

During SWS, sharp wave/ripple complexes occur in the hippocampus. Sharp wave/ripple complexes are fast depolarizing events (sharp waves) overlapping high frequency local field potential oscillations (ripples) originating from pyramidal neurons in the CA1 region of the hippocampus [68-71]. Replay is nested within sharp wave/ripple events. Ripples themselves may provide a memory function. For example, the frequency of ripples (150–250 Hz) is suitable to spawn long-term potentiation [72]. Moreover, Girardeau and colleagues [73] demonstrated that experimentally suppressing hippocampal ripples impairs memory. In this experiment, rats were trained to find chocolate cereal in a radial arm maze. During subsequent sleep, stimulation to the hippocampus selectively disrupted the occurrence of ripples. As a consequence, spatial memory for the location of the reward in the armmaze was impaired in these animals relative to a group of animals that received stimulation that did not disrupt ripples and a nonstimulation control group.

The density of ripples is also modulated by learning, further supporting a mnemonic role of ripples. The number of sharp wave/ripple events and their duration increase immediately following learning [74]. Conversely, animals that fail to learn a given task do not show a change in the sharp wave/ripple event characteristics. In fact, ripple density directly correlates with subsequent performance. This was demonstrated by Ramadan and colleagues [75] who, using a similar spatial memory task as Girardeau and colleagues [73], found that animals with the greatest ripple density in sleep following learning made the fewest errors in subsequent recall of the spatial memory task.

Rather than just triggering local long-term potentiation and replaying within the hippocampus, slow wave/ripple events are associated with transfer of memories from temporary storage in the hippocampus to more permanent storage in the cortex. This two-stage process of memory, moving from temporary to long-term storage, is consistent with memory models which suggest that such a process is essential for memories to be formed without damaging existing memories and causing hallucinations [76]. Moreover, lesion studies support the role of hippocampal structures in temporary, short-term memory storage [77]. Such temporally graded amnesia is not found with neocortical lesions, suggesting that neocortical areas contribute later in the life of the memory [78].

Sharp wave/ripple events are associated with the occurrence of sleep spindles in the neocortex [79]. In addition to a role in sleep maintenance [25, 26], sleep spindles are considered markers of brain plasticity [80, 81]. Moreover, Schmidt and colleagues [82] found an association between learning and spindle density. Specifically, human participants learned lists of word pairs. In one condition, the words were concrete (e.g., APPLE-CHAIR) making them more memorable than the other list that was composed of abstract word pairs (e.g., UNION-RATE). Spindle density was increased in a subsequent nap relative to a baseline nap following learning of the more difficult list of abstract word pairs but not following learning of the simpler concrete word pairs.

Thus, ripples are likely to promote learning in the hippocampus while spindles provide likewise to the cortex. But rather than working independently, slow oscillations bind together the content of ripples and spindles. Ripples occur within spindle troughs [79] while spindles are aligned with slow oscillations [83]. Slow oscillations in SWS are initiated over the frontal lobes but spread to excitatory and inhibitory neurons across the neocortex [84, 85]. Importantly, however, hippocampal activity precedes cortical activity, consistent with the theory that memories are transferred from hippocampus to neocortex [79, 86].

Marshall and colleagues [87] demonstrated the role of slow oscillations in human memory consolidation. By applying transcranial direct current stimulation with an oscillating frequency of .75 Hz during early NREM sleep, naturally occurring slow oscillations were enhanced. Prior to sleep, participants learned word pairs and recalled these pairs after sleep. Significantly more pairs were recalled following sleep with oscillation-boosting simulation than in a condition without stimulation.

Thus, while some have argued against an active role of sleep in memory via consolidation [9, 88, 89], neurophysiological studies support such a role of sleep in memory. If sleep merely passively protected memories from interference from ongoing sensory input, many of these associations with sleep physiology would not be expected. Rather, cued reinstatement of memories during sleep and enhanced performance following induced slow oscillations provide powerful evidence in support of sleep’s active memory function.

## 5. Sleep-Dependent Generalization

While the scope of this paper so far is sufficient to explain why declarative memories are more accurately recalled in their literal, veridical form after sleep than they are after wake, it is unaccounted for is how sleep benefits other cognitive processes such as decision making [11] and creativity [9]. For example, we demonstrated that decisions are more optimal following sleep than following wake using a variant of the Iowa Gambling Task [11]. In this task, participants are presented with four decks of cards and the win/loss likelihood of the cards in each deck varies such that two decks are “good” and two decks are “bad” [90, 91]. We gave participants brief exposure to each of the decks (6 computer-guided draws from each deck) either in the morning or the evening. Following a 12 hr interval spent awake or a 12 hr interval containing overnight sleep, participants freely drew cards with the goal of “winning the most money.” We found that participants in the sleep group made more optimal draws (choices from the “good” decks) than those in the wake group. A comparison of performance for a “morning” group, who performed the entire task in the morning, and an “evening” group, who performed the full task in the evening, revealed no group differences. Therefore, the optimal performance of the sleep group relative to the wake group cannot be accounted for by circadian fluctuations in performance [11].

An interval with sleep also enhances the extraction of the “gist” from a list of memorized items. This has been demonstrated repeatedly using the Deese-Roediger-McDermott paradigm, a paradigm that was designed to study false memory formation [92]. In this task, participants are presented with lists of words to learn. Words on each list are semantically related (e.g., BOWL, SPOON, and MILK) but lack a critical lure that reflects the gist of the word list (e.g., CEREAL). When participants encode these lists prior to an interval with sleep, recall of the items on the list 12 hrs later is greater than when recall follows wake, consistent with sleep’s benefit on veridical memories as previously described. Interestingly, the number of critical lures recalled is also greater following sleep than wake [93-95]. In other words, following sleep, participants are more likely to report a word that was never on the original list but that captures the gist of the list.

Recently, Lau and colleagues [96] taught English-speaking individuals sets of Chinese characters that shared ideographic components. These shared ideographs, or similarities in the structure of the Chinese character, are found for many semantically related words (e.g., SISTER, PRINCESS). Participants who napped after learning these sets of characters performed better on a subsequent test of veridical memory for the learned characters compared to a group who stayed awake after learning. Moreover, just as in the Deese-Roediger-McDermott studies, the nap group also improved more in a measure of learning of novel characters that shared ideographics with the original set (e.g., MAID, MOTHER). This nap benefit was observed both when the nap took place immediately and when the nap took place 90mins after encoding.

To perform optimally in the Iowa Gambling Task or to recall a critical lure in the Deese-Roediger-McDermott paradigm, requires inferring generalities from veridical knowledge. In a direct study of inference, Ellenbogen and colleagues [97] reported evidence of sleep’s role on transitive inference. In this study, participants were presented with pairs of abstract images (e.g., A-B, B-C, and C-D). Through forced-choice, participants learned a “correct” item in each pair. Unbeknownst to the participants, the value of items within a set was determined by its position within a hierarchy (e.g., A>B>C>D). At test, participants were presented with never before seen pairs (e.g., A-C, B-D). By inference of A’s and C’s relationship to B in this example, participants can choose the correct item even in these never before seen pairs. Shortly after (~20 mins) encoding of the base pairs, participants were at chance in solving inferential pairs. Yet, Ellenbogen found that participants that slept after viewing the core pairs (e.g., A-B, B-C) were better at solving these inferential pairs (e.g., A-C, B-D) 12 hrs later than those participants that were awake in the intersession interval. Moreover, those that slept were even able to resolve pairs that required inference across great distances (e.g., A-D).

In a similar vein, Durrant and colleagues [98] described this inference function as statistical extraction. They examined participants’ abilities to recognize completely novel sequences of tones based on their statistical similarity to previously learned tone series. Consistent with a role of sleep in generalization from veridical memories, the authors found that novel tone sequences that were statistically similar to learned sequences were better recognized following both overnight sleep and a mid-day nap relative to equivalent intervals of wake. Again, sleep had an effect not simply on the literal memory of the original sequences but improved performance on never before heard sequences through a process of statistical inference.

These examples all capture what may be more broadly characterized as generalization. Generalization is “*a process that allows organisms to build on prior experience and respond flexibly to new information outside of the context in which a memory was initially formed* [99].” Generalization of veridical knowledge underlies inference, statistical extraction, and gist extraction. Here we introduce the term sleep-dependent generalization to refer to the generalization of veridical learning that preferentially occurs over sleep.

A series of studies by Gomez and colleagues [100, 101] demonstrated sleep-dependent generalization in infants. Infants were presented with auditory nonword strings (e.g., PEL-WIFFLE-RUD), an artificial language learning paradigm thought to mimic real language learning. Infants who slept less than 30mins in the intervening 4 hr interval (“wake” group) demonstrated veridical memory for the nonword strings as measured by increased looking time in the direction of learned auditory strings relative to looking time corresponding to novel strings. However, infants who slept more than 30mins in this 4hr interval (“sleep” group) were biased by the first string presented. If the first string was familiar (from the learning phase), they showed a preference for familiar strings; if the first string was unfamiliar (but statistically similar to familiar strings), these rested infants preferred unfamiliar strings. This was taken as evidence of generalization of the grammar rules underlying the artificial language. Importantly, the benefit of having napped on generalization was still present the following day suggesting that group differences were not due to differences in alertness or emotional arousal in the wake (napless) group [101]. Thus, this series of studies supports a role of sleep-dependent generalization in early development.

Recently, we demonstrated how sleep-dependent generalization has a translational benefit, specifically in the treatment of anxiety disorders such as spider phobia [102]. When treating such disorders, psychotherapists often present examples of the feared cue (e.g., spiders) to the patient with the assumption that exposure will diminish the fear response. A weakness of this treatment is that the reduced fear response to exemplars does not generalize to the nonexposed spider outside of the treatment context. In our study, individuals with spider phobia were presented with short videos of spiders. Following overnight sleep or daytime wake, participants were presented with videos of the old spider as well as novel spiders. Based on subjective (disgust, fearfulness, and unpleasantness) and objective measures (corrugator muscle activity, skin conductance response, and heart rate), fear of the exposed spider decreased across the experiment. However, those that slept following initial exposure showed better retention of the reduced fear to the exposed spider and, importantly, better generalization to a novel spider relative to a group that spent the intersession interval awake. These results suggest that exposure therapy may be most effective if timed such that sleep may occur afterwards thereby allowing the learned fear extinction to be generalized to novel stimuli.

In sum, sleep-dependent generalization has gained wide support from the behavioral literature. This function may underlie early learning and has translational significance. For this reason, it is useful to understand the neurophysiological basis for this sleep function.

## 6. Neurophysiology of Sleep-Dependent Generalization

Generalization over sleep cannot be explained by simple neural replay or spindle induced cortical plasticity. In the case of spider exposure therapy; for instance, neural replay would reinforce the veridicalmemory for the exposed spider but this mechanism should not necessarily enhance the memory for (or decrease reactivity to) an unexposed spider. While little research has been done on the physiological basis for sleep-dependent generalization, several studies collectively suggest a viable mechanism. Specifically, generalization may occur through “replay” of novel sequences.

Several studies have observed replay in which the sequence of neural firing was in reverse order from the sequence recorded during recent exploration [103, 104]. While forward and reverse replay can be found in succession, Diba and Buszáki [104] found that forward replay accounted for the majority of the sequences recorded shortly before the animal began a new navigation, or “preplay” of the future sequence. These results suggest that hippocampal replay may be adapted to serve both a preparatory role (forward preplay in anticipation of an experience) and an exploratory role (recently explored sequences via reverse replay).

While reverse replay alone may contribute to some generalization, a recent study by Gupta and colleagues [105] suggests an even more powerful role of replay in generalization. In this study, rats were trained on a two-choice maze. Regardless of the most recently traversed paths, the neural ensemble associated with both rewarded maze paths were replayed during the subsequent rest period. However, what is most striking is that some neural ensembles recorded during rest were associated with never experienced shortcuts. In other words, at the level of the hippocampal place cells, the animal was considering novel solutions that were derived from the known solutions to the maze.

Neural replay is most often associated with hippocampal CA1 place cells that form the neural map of space. As such, translation of these results with respect to spatial memory is obvious: sleep-dependent generalization should lead to a novel route to take home from work or to get from New York to Chicago. But how are semantic generalization and concept abstraction achieved? To answer this, consider that replay is not isolated to hippocampal place cells. Rather, replay is also observed in the cortex. For instance, ensembles associated with waking experiences are reactivated in posterior parietal cortex [106] and the primary visual cortex (V1) [107]. Whether neocortical replay outside the hippocampus has the potential for creative recombinations is not known. But rather than simply altering the sequence to drive generalization, novel concept combinations may be activated simply through the reactivation of overlapping concepts during replay. According to Lewis and Durrant’s [108] Information Overlap to Abstract (iOta) model, reactivation of two memories that share an overlapping concept will result in strengthening the areas of overlap, which may represent the “gist.”

A recent computational memory model lends support to the suggestion that recurrent activity in hippocampal loops may underlie sleep-dependent generalization. In the model, Recurrency and Episodic Memory Results in Generalization (REMERGE), Kumaran and McClelland [109] suggest that generalization arises from the replay of recent memories in hippocampal CA1 cells. But rather than merely replaying the ensemble associated with the recent experience, the model suggests that multiple related conjunctive units are also activated, a mechanism proposed to support generalization. In fact, a simulation of the transitive inference study of Ellenbogen and colleagues [97] using REMERGE was able to replicate the failure to solve inferential pairs 20 mins after encoding and the emergence of accurate inference performance following 12 hrs with sleep [109].

As with veridical replay which takes place during SWS, Gupta and colleagues [105] observed that the neural firing was associated with never-experienced shortcuts also during SWS. This is consistent with behavioral observations of sleep-dependent generalization in SWS. For instance, the over-sleep change in performance on the probabilistic tone sequence learning task was associated with time spent in SWS. Specifically, those participants who spent the greatest proportion of sleep in SWS had the greatest postsleep performance [98].

However, others have proposed that generalization comes about over REM sleep. Walker and Stickgold [14] proposed that REM sleep supports memory unitization (the binding of elements into a single representation), memory assimilation (the integration of new information into existing concepts or schema), and memory abstraction (the isolation of concepts or schema from new information). Abstraction is presumably the same function referred to here as generalization. According to Walker and Stickgold, explicit representations from prior waking are consolidated over SWS. During subsequent REM sleep, these primed memories are then generalized via theta wave facilitated corticocortical processing in association areas with minimal hippocampal or dorsolateral prefrontal cortex contributions. The authors turn to behavioral evidence from two tasks. First, performance on the Remote Associates Task is associated with REM sleep [9]. In this task, participants are given a list of unrelated words (e.g., COOKIES, SIXTEEN, and HEART) and are required to find the word that underlies the association between them (e.g., SWEET). Participants were equally capable of finding the critical association following a nap and quiet wake. However, if participants were given an implicit prime (solving analogies that contain solutions to the Remote Associates Task), participants were able to generate more critical associations in the Remote Associates Task but only if they had REM sleep in the intervening nap. Second, REM sleep is associated with impaired performance on a logic task (Wff “n” Proof) [110]. In this study, participants learned the rules of the task and performance was tested again 8 days later. REM sleep was reduced in the sleep following learning in a subset of individuals by ingestion of alcohol prior to sleep. These REM-impaired individuals had less sleep-related performance improvement than those with normal REM.

A parsimonious alternative is that SWS and REM play a sequential role in memory consolidation and generalization. Recent studies exploring the synaptic homeostasis hypothesis shed light on this possibility. According to the synaptic homeostasis hypothesis proposed by Tononi and colleagues [111, 112], synaptic potentiation, which increases over wake, is decreased over sleep. In fact, without such downscaling of synaptic weights, the brain would literally run out of capacity. As described by Tononi and Cirelli [111], “…*due to the combined energy and space costs of uninterrupted synaptic plasticity, the ability of the brain to acquire new information would rapidly grind to a halt in the absence of downscaling*.”

Electrophysiology studies in the rat support the synaptic homeostasis hypothesis. Vyazovskiy and colleagues [113] measured AMPA receptors with the GluR1 subunit in rats following periods of sleep and wake. Long-term potentiation (LTP), a well-known cellular mechanism of memory formation, is associated with delivery of GluR1-containing AMPA receptors to the synaptic membrane. Thus memory, in part, reflects increased excitatory receptors making future stimuli generate quicker and larger responses [114, 115]. Rats that were awake for the majority of a 6 hr dark period (rats are nocturnal) had high levels of GluR1-containing AMPA receptors while rats that were asleep during the majority of a 6 hr light period had low levels. Moreover, the cortical evoked response, local field potential responses to electrical stimulation that serve as a proxy for synaptic efficacy, was increased after wake and decreased after sleep. This result provides support for the simple predictions of the synaptic homeostasis hypothesis that synaptic potentiation increases over wake and decreases over sleep.

Direct measurement of synaptic density and size in *Drosophila* brain provides further support for the synaptic homeostasis hypothesis [116]. Synapse density and size increase over wake. This increase is associated with experience given that synaptic growth is greater following enriched wake than after periods of unenriched wake. Specifically, flies who spent 12 hrs in a “fly mall,” allowed to freely move around and interact with up to 100 other flies in a chamber, had greater synaptic spine density than flies who spent the 12 hr interval housed individually, which is considered to be an unenriched environment. Next, Bushey and colleagues had flies that had the luxury of the fly mall subsequently housed in individual tubes for 7 hrs and either allowed them to sleep or sleep deprived them via mechanical stimulation. They found that following sleep deprivation, spine size and density were unchanged. However, following sleep, spine size and density regressed. In fact, synaptic morphology was equivalent to that of flies housed individually, having never experienced the joys of the fly mall.

While synaptic downscaling following sleep has been repeatedly demonstrated, the mechanism and timing of synaptic downscaling is uncertain. Widespread oscillations between polarization and depolarization, the slow wave oscillations found in SWS, are ideal for generating depotentiation. Thus, is maintained over sleep via synaptic downscaling, specifically during SWS [112].

However, a series of recent studies have failed to support the prediction of synaptic downscaling over SWS. Chauvette and colleagues [117] recorded local field potentials in somatosensory cortex in response to stimulation of the medial lemniscus (i.e., cortical evoked response) before and after SWS. If synaptic weight is reduced over SWS, the cortex should be less excitable. In other words, stimulation of the medial lemniscus should evoke a smaller response in the somatosensory cortex following sleep compared to before sleep. To the contrary, Chauvette and colleagues [117] found an *increase* in cortical evoked responses following SWS compared to prior waking.

A parallel study suggested a role of REM in achieving synaptic downscaling. Grosmark and colleagues [118] took the approach of recording local field potentials in hippocampal CA1 cells during NREM and REM sleep and subsequent wake. While local field potentials do not directly measure synaptic potentiation, changes in global firing rate across the hippocampus would largely be expected to decrease in conjunction with downscaling. Counter to predictions, excitability increased over SWS. Rather, excitability decreased over REM sleep and this reduction was greater than the SWS increase, resulting in a net decrease in excitability over sleep. This result suggests that NREM and REM sleep are both important to the overall maintenance of synaptic homeostasis. Moreover, Grosmark and colleagues [118] found increased ripple-related firing across NREM sleep epochs. Interestingly, this increase was most prominent in the neural ensembles with the greatest theta and gamma activity during intervening REM. Collectively, these results led Born and Feld [119] to speculate that global downscaling over sleep as a whole is accompanied by local upscaling over NREM-REM sequences.

In sum, there is ample support for the synaptic homeostasis hypothesis. Synaptic potentiation is reduced across the brain over sleep. Importantly, this process may not occur in a single, SWS-dependent step but may rely on REM-SWS sequences. We will return to the interaction between SWS and REM in Section 11.

## 7. Selective Remembering and Forgetting over Sleep

Francis Crick, best known for his part in the Nobel prizewinning discovery of the DNA helix, also had a fascination with neuroscience and, specifically, memory. This “hobby” led Crick and his colleague, Graeme Mitchison, at the Sulk Institute to a largely theoretical proposal suggesting that sleep benefits forgetting [120]. At first light, this hypothesis seems completely counter to work reviewed so far, which supports the fact that we sleep to remember. However, Crick and Mitchison describe sleep as playing a role in selective forgetting. They suggest that the interconnected neural networks are susceptible to unwanted, or “parasitic,” connections and these may be detected and pruned over sleep.

This “sleep to forget” hypothesis remained largely theoretical until very recently when a series of behavioral studies have tested its validity. Forgetting has been studied in the psychological literature with two paradigms, retrieval induced forgetting [121] and directed forgetting [122], both of which have now been examined with respect to sleep. The retrieval induced forgetting paradigm implicitly induces forgetting of a conflicting subset of items in favor of memory for a practiced subset. Specifically, participants encode a list of word pairs. Word pairs are composed of targets paired with six associated words (e.g., EGG-BACON, EGG-TOAST, EGG-CHICKEN, EGG-EASTER, EGG-OMELET, and EGGYOLK). Participants practice recalling word pairs with cues directing them to recall half of the original pairs (e.g., EGG-B__, EGG-T__, and EGG-C__). Previous studies have demonstrated that recall of the practiced pairs is enhanced in conjunction with forgetting of unpracticed pairs (e.g., [121, 123]). We examined sleep’s role in remembering and forgetting by having participants either sleep or stay awake following the encoding and retrieval practice phases of this task [124]. Consistent with data reviewed above, sleep-dependent consolidation of the practiced pairs was observed. That is, pairs that participants practiced recalling (e.g., EGGBACON) were better recalled by participants who slept following encoding compared to recall by participants who stayed awake following encoding. Surprisingly, we also found that those pairs that were intended to be suppressed or forgotten by their overlapping association with practiced pairs (e.g., EGG-YOLK) were also better recalled after sleep than after wake. This pattern of results was found both over an interval of overnight sleep compared to daytime wake and over a daytime nap compared to an equivalent interval of daytime wake, thus excluding a circadian explanation for the results.

Unlike the retrieval induced forgetting paradigm, the directed forgetting paradigm uses an explicit approach to forgetting. Participants are presented with words and, after a brief interval (e.g., 500ms), are instructed to “remember” or “forget” the previous word. A number of studies have demonstrated that participants are more accurate in recalling to-be-remembered words compared to to-be-forgotten words (for review see [125]). Using this approach, Saletin and colleagues [126] suggested that sleep does, in fact, enhance forgetting. Immediately following encoding, to-be-forgotten words were recalled less than to-be-remembered words. This distinction between instruction types was still evident following a 6 hr mid-day interval both with and without a nap. Unsurprisingly given the literature reviewed so far, the nap group recalled more words, regardless of instruction type, than that of wake group. However, the difference in recall between the nap and wake groups was driven by greater recall of to-be-remembered words in the nap group relative to the wake group. There was no difference in recall of to-be-forgotten words across groups.

While these studies of sleep’s role in forgetting appear to conflict, a parsimonious explanation is that the significant differences in the motivation to forget underlie the apparent contradiction. Specifically, in the directed forgetting paradigm, participants are clearly instructed as to the future relevance of a subset of stimuli. A to-be-remembered cue has future relevance, as the participant is aware of the need to recall it later. A to-be-forgotten stimulus lacks such future relevance; rather these stimuli are clearly irrelevant. In the retrieval induced forgetting paradigm there is no clear reason, at an explicit or implicit level, why one would not want to remember both practiced and unpracticed pairs. Certainly, if memory must make sacrifices, the practiced pairs are stronger and will be remembered. But given sufficient capacity, all should be remembered. Thus, in the retrieval induced forgetting paradigm, practiced and unpracticed pairs have future relevance and, as such, are consolidated over sleep while in the directed forgetting paradigm, sleep protects only items with instructed future relevance while sacrificing those without future relevance.

The role of sleep in selectively remembering items with future relevance has been directly examined. Wilhelm and colleagues [127] had participants learn a list of word pairs. After encoding, half of the participants were explicitly told that they would be asked to recall the word pairs following a 9 hr break, which was filled with daytime wake, nighttime sleep, or nighttime wake. Recall following the break was the greatest for those individuals who slept and were aware of the subsequent need to recall the pairs. Those individuals who were unaware that they would be asked to recall the word pairs following the break did not have significant performance enhancements compared to the wake groups. Thus, this study directly demonstrates sleep’s selective role in memory consolidation, acting only on those memories that have future relevance.

Emotion provides another source of future relevance. It is evolutionarily advantageous to avoid items that pose a threat. As such, negative emotional images or events are important to remember and for this reason, a negativity bias is typically observed in studies of memory encoding and recall [128]. Sleep has been shown to consolidate and, in some cases, preferentially enhance memory for negative emotional images relative to neutral images [129-132]. Emotional reactivity associated with negative images is also preserved over sleep [133].

Collectively, these studies illustrate that sleep-dependent consolidation is selective. Declarative memories are not uniformly consolidated over sleep. Rather, memories with future relevance, either for their rewarding (doing well on the forewarned delayed recall) or protective (steer clear of a person who is a threat) influence, are preferentially maintained over sleep.

## 8. Neurophysiological Basis of Selective Memory and Forgetting over Sleep

To selectively consolidate memories with future relevance and suppress those without, assumes that the brain has a mechanism for such categorization of memories. One well-known form of brain-based classification is the “tagging” of emotional memories by the amygdala. As stated above, emotional memories (particularly those with negative affect) are better remembered than neutral memories. The emotional tagging theory posits that by activating the amygdala when a memory is encoded, associated synapses are tagged (e.g., reduced threshold for activation) making them more susceptible to consolidation later [134]. The amygdala is reactivated during REM sleep. This, in conjunction with favorable physiological and neurochemical conditions, has led some to propose that emotional memories are processed over REM sleep. Supporting this conjecture, recognition memory for negative images correlates with time spent in REM sleep [130]. Moreover, we found a negative correlation between the over-sleep change in subjective valence of negative images and time spent in REM sleep.

Yet, the behavioral data reviewed above suggests that nonemotional, nonamygdala activating memories can also be parsed for future relevance during consolidation. A study by Poe and colleagues [135] suggests that this, too, may be a function of REM. In this study, hippocampal cells were recorded while rats traversed familiar or novel environments. In subsequent REM sleep, a clear distinction was found in the phase locking of theta with firing of cells associated with novel versus familiar environments.

Thus, physiological data suggest that memories may be sorted during REM. Whether synaptic tagging by the amygdala is completely distinct from the theta dissociation for events with future relevance is not yet clear. In Section 11 we will return to this issue, discussing how segregation in REM relates to memory consolidation in SWS.

## 9. The Role of Sleep in Consolidation of Motor Skill Learning

To this point, this paper has focused on the consolidation and generalization of nonmotor, declarative memories. Pioneering studies with patient HM, who had severe amnesia as the result of the removal of the hippocampus to treat epileptic seizures, were the first to neurally dissociate motor skill learning from declarative learning. While HM was known to have massive deficits in episodic and semantic memory [136], Milner [137] noted his preserved ability to learn a mirror-tracing task. In this task, the participant is given a template outlining a figure (e.g., a star) and the participant must trace this outline viewing their hand and the trajectory only through a mirror, requiring visuomotor adaptation. In years since a taxonomy of memory has been established that largely distinguishes declarative learning (learning of facts and events) from motor skill learning and other forms of procedural learning [138].

A number of studies have demonstrated that motor skill learning is benefited by sleep. This was made clear in a seminal paper by Walker and colleagues [139] who examined performance on a simple finger sequence learning task before and after intervals with wake and sleep. Participants were given a sequence (e.g., 4-3-1-2-4 where the numbers cue a specific finger or key) and were told to press this sequence of keys as quickly as possible for 30 sec blocks. Following an intersession interval awake, performance did not improve significantly. However, following sleep, participants were 18–20% faster, a sign that learning of the sequence had taken place. While subsequent studies have emphasized that over-sleep performance changes may not reflect enhancement per se [140, 141], sleep-dependent stabilization of performance has been replicated across many studies [142-145].

Likewise, the mirror-tracing task that HM proved to be able to learn [137] is benefited by sleep [146-148]. In one such study, Nissen and colleagues [149] had participants trace figures prior to sleep (e.g., 8 pm) or prior to a daytime wake (e.g., 8 am). While there was no group difference in initial performance, ruling out circadian influences on this task, when performance was probed 12 hrs later, improvements in speed and accuracy were greater for the group that slept between sessions. Interestingly, a group of individuals with primary insomnia, a disorder characterized by difficulty falling and staying asleep, did not show any sleep-specific improvements in performance.

There are notable exceptions to the observation of beneficial effects of sleep on procedural learning. For example, in a visuomotor adaptation task in which participants learned to make reaches with an inverted-map joystick, performance changes over sleep and wake were equivalent. This result is particularly surprising given the similarity to mirror tracing which is preferentially consolidated over sleep [146, 147]. Nemeth and colleagues also failed to demonstrate sleep-dependent memory consolidation on a probabilistic motor sequence learning task in which random items are interleaved in between an 8-item sequence (e.g., 1-R-2-R-3-R-4- R where 1–4 are specific response keys and “R” represents a random response key). Understanding the neurophysiological basis for procedural memory consolidation over sleep may help in understanding the discrepancy in these reports.

## 10. Neurophysiological Basis for Consolidation of Motor Skill Learning over Sleep

Motor skill learning is rarely studied in the small animal models that are commonly used in sleep neurophysiology studies. For this reason, a clear distinction in neurophysiological processing of nonmotor and motor learning over sleep has not been examined. However, from the available literature, we can conjecture as to how motor skill learning might consolidate over sleep.

It is worth considering whether procedural memories may be consolidated via replay in the subcortical structures engaged in the learning task. In fact, neural replay during sleep has been found in ventral striatum [150]. However, importantly, this activation is led by hippocampal reactivation of related place cells [151]. Whether striatal or even cerebellum-based replay occurs in the absence of hippocampal reactivation is unexplored.

Nonetheless, a hippocampal-striatal replay mechanism should not be excluded as a mechanism for motor skill memory consolidation. In spite of HM’s spared ability, many forms of motor skill learning are now thought to engage the hippocampus after all. This view is supported by a recent corpus of literature that suggests that the hippocampus is engaged in many forms of motor learning, including many forms of motor sequence learning [152, 153]. As such, we have predicted that sleep will benefit the retention of motor skills that engage the hippocampus.

Whether a motor skill learning task engages the hippocampus is likely to depend on a number of factors. First, explicit awareness of what is being learned in a procedural task is likely to engage cognitive control utilizing hippocampal resources [154]. Second, when motor skill learning requires the binding of information across dimensions, the hippocampus is known to be engaged. Supporting this relational memory theory of hippocampal function [155] is evidence that the hippocampus binds faces to names [156], objects to scenes [157], and landmarks to cities [158]. Third, if a procedural task is rewarded, this too is likely to draw hippocampal involvement. Long-term retention of procedural learning is enhanced when learning is rewarded [159] an effect proposed to be driven by dopaminergic modulation of the hippocampus.

We tested whether hippocampal engagement at encoding might predict the presence of later sleep-dependent consolidation of motor skill learning. We contrasted three task variants: explicit motor sequence learning, when the participant is aware of the sequence being learned; implicit motor sequence learning, in which the participant is unaware of the sequence being learned; and a contextual motor sequence learning task which places spatial sequence in the context of a color sequence yet the participant is unaware of either sequence [160]. Based largely on neuroimaging studies, explicit and implicit contextual forms of sequence learning have been shown to engage the hippocampus at encoding while noncontextual implicit sequence learning does not [161]. We found sleep-dependent performance improvements on the hippocampus-engaging variants of the task, explicit and implicit context sequence learning, but not on the implicit variant which does not engage the hippocampus [160].

Thus, we propose that the level of engagement of the hippocampus during encoding can account for discrepancies in the literature regarding consolidation of motor skill learning. For instance, the implicit probabilistic motor sequence learning task is designed such that sequence awareness is deliberately prohibited and contextual associations (such as between items) are not possible (see [160, 161]). As such, the lack of sleep-dependent consolidation observed by Nemeth and colleagues [162] may be explained by hippocampal disengagement. Likewise, differences in awareness in the visuomotor task, adopted by Doyon et al. [163] may distinguish it from the mirror-tracing tasks in which sleep has been deemed beneficial [146, 147].

## 11. An Integrated View of the Neurophysiological Basis of Sleep’s Function on Memory and Cognition

While we have reviewed these sleep functions and underlying physiological mechanisms individually, an integrated view of the neurophysiology underlying vast behavioral benefits of sleep on learning and cognition is becoming clear. In this section, we attempt to lay out this view. While this proposal is not completely unique (e.g., see [108, 119, 164-166]), we conjoin features of various models with recent data to provide a more integrated account than what has previously been possible.

**Figure 1:**
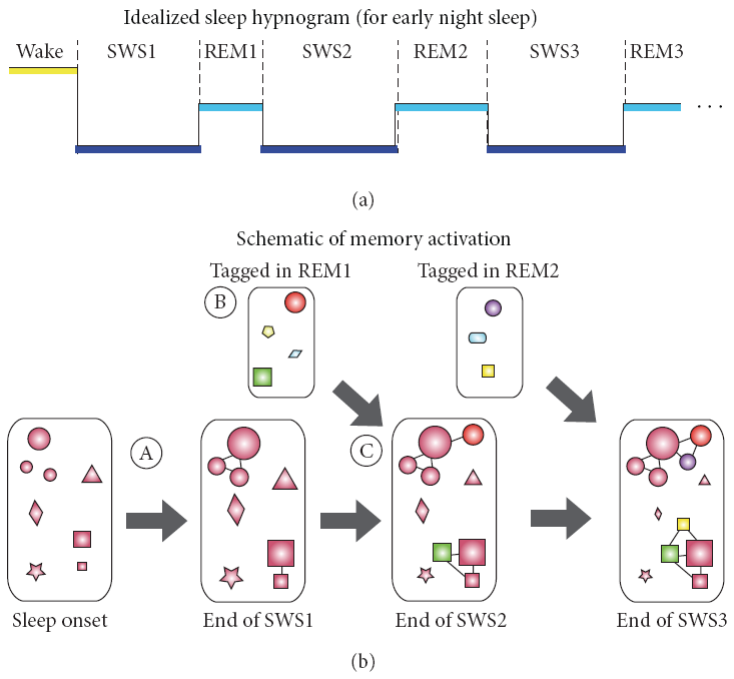
Model of the neurophysiological basis of sleep’s function on memory and cognition. Brain states cycle throughout the night between nREM and REM sleep stages. Early in the night (a), SWS predominates with interleaving bouts of REM sleep. The evolution of the memory varies across these cycles (b). At the beginning of the night (A), recent memories (represented by various shapes) are active. In early SWS, overlapping recent memories (illustrated as circles and squares) are integrated. During interleaving REM (B), similar existing memories (circles and squares) and new memories with future relevance are tagged for integration in subsequent SWS bouts (C).

As idealized in Figure 1, human sleep typically begins with SWS (following brief epochs of light NREM, NREM1, and NREM2). We can only speculate as to the cause of the increase in global synaptic potentiation as evidenced by the increased excitability reported by Grosmark and colleagues [118]. But one possibility is that wake ends with high potentiation of recent and salient memories, and these memories as well as memories efarlier in the day are increasingly potentiated in early SWS. At this time, overlapping schema from the day can be integrated through simultaneous activation as proposed by the iOta model [108] resulting in local depotentiation as distinct episodes are bound (Figure 1(a)).

Bouts of REM sleep are interleaved between SWS bouts. As reviewed above, in REM memories are distinguished based on future relevance and, possibly, relatedness to memories with future relevance (Figure 1(b)). At the same time, downscaling (decreased excitability) over REM [118] may come about from decreased activation of memories deemed irrelevant or with low emotional value (reduced size of star, diamond, triangle across Figure 1).

In SWS that follows REM, memories tagged during REM are integrated with recent memories, particularly those nodes strengthened by local integration in the prior SWS epoch. The activation associated with recent memories in conjunction with relevant past memories again results in a global increase in synaptic weight, but binding of related memories results in areas of local downscaling.

Presumably this SWS-REM cycle could continue throughout early sleep. In humans, SWS is greatly reduced in the last 50–70% of the night. Rather, REM cycles with NREM2. There is evidence that in this latter part of sleep, with declarative memories sufficiently consolidated, processing of motor memories begins to be predominant [139, 147]. Oversleep changes in motor skill learning correlate with time spent in late NREM2 sleep [139] and NREM2 spindles [147]. NREM2-REM cycles may behave in a similar way to the picture in Figure 1 but changes in activity shift to motor-based memories.

Other aspects of this model can be supported by correlations between sleep stages and behavior as well. Sleep-dependent consolidation of simple declarative learning tasks is correlated with time spent in SWS [167-169]. However, by this account emotional memory consolidation would require both REM (for sorting memories) and SWS (for consolidation). Consistent with this, emotional memory consolidation [130] and the protection of the emotional reactivity [133] correlate with time spent in REM as well as time in SWS [170]. Likewise, memories that are filtered for nonemotional future relevance should benefit from SWS-REM sequences. Here, too, there are mixed reports of advantages of both SWS [127] and REM [124]. While we would predict both to be true in any given account, few studies consider a sequential benefit in such correlations (but see [171]) and there are many complexities that may make this difficult to elucidate with this correlational approach.

We admit to several gaps in the framework laid out here. Nonetheless, by drawing together what is known in such a way, testable hypotheses can be derived in order to educate these gaps.

## 12. Conclusions

While sleep is a period of bodly rest, it is also a period of brain activity and an opportunity for cognitive function that we take for granted. Marked changes in neural activity and the neurochemical amalgam allow for cognitive processing to occur in ways that are not possible over wake. Selective memory consolidation and generalization of declarative, procedural and emotional memories have vast impacts on waking cognitive performance. Decisions are improved by sleep-dependent generalization [11, 12, 172]. One might imagine that the flood of product descriptions viewed when online shopping in the evening is integrated with our stored memories to isolate the ideal features based on the future uses the product might serve. Meanwhile, the minutia of our day is filtered out, making room for emotional memories and academic material that will come in handy later (e.g., [173]). Simultaneously, we can generate creative ideas upon awakening as the result of combining memories over sleep. Such combinations have brought about the discovery of benzene’s ring-like shape, the tune to The Beatles’ “Yesterday”, and Percy Shelley’s *Frankenstein*—all of which have been said to come from dreams [174]. Collectively, the function of the sleeping brain should entice us to sleep.

Progress in understanding how these great achievements come about over sleep has been rapid over the past decade. As the present model and other models are tested and honed, we may better understand how impairments in sleep account for learning and cognitive deficits in developmental populations and clinical populations. Treating sleep may improve cognitive function, and conversely, enhancing daytime cognitive activities may provide a similar wealth of benefits to subsequent sleep.
